# Caseous Degeneration

**Published:** 1877-07

**Authors:** Geo. E. Walton

**Affiliations:** Cincinnati, O.


Caseous Degeneration.—*By Geo. E. Walton, M.D., Cincinnati, O.*—An exceedingly necessary condition of the consideation of this subject is a knowledge of what is meant by the term "caseous degeneration." As far as my investigations of the subject go, it is this: A disease of cells in which the protroplasm of the cell is transformed into fat; a deposit therein of saline substances takes place, and the fluids of the cell are in great part removed. As a consequence of this deposition this form of degeneration can occur in any part of the body where there are cells; but some tissues are especially subject to it—thus the digestive, respiratory and genito-urinary mucous membranes, the pulmonary alveola, also serous surfaces and within the texture of certain organs. The circumstances with which the occurrence of caseous degeneration is associated, are chronic inflammations—inflammations of a so-called low grade.

It is exceedingly questionable whether pus ever takes on caseous degeneration. Says Billroth: "When these yellow spots are found in the cadaver, it is often supposed that they correspond to a dried collection of pus; but this is not true, or at least very rarely so. Most of the cheesy collections were from the first in miniature what they now are in gross, and were never fluid pus. Those who maintain the conversion of pus into caseous matter state that it occurs when the pus is pent up or confined for a long time within deep structures. It is well, however, to bear in mind that the mere fact that the pus is so confined is an evidence of chronic inflammation—an indication of that condition of the system in which the products of imflammation are prone to resolve themselves into caseous matter instead of pus; and it is not impossible to conceive that cells, on the one hand, may undergo caseous degeneration, and, on the other, conversion into pus—the two processes marching into line, without the deduction that the one has been transmuted into the other.

Since caseous degeneration is distinguished by the conversion of the protoplasm of the cell into fat, we ask what is the difference between caseous degeneration and fatty degeneration? It seems to be this—that when a cell becomes affected with fatty degeneration, the protoplasm and entire contents thereof become converted into oil; in the place of the cell we have an oil globule. In the fatty degeneration of muscular fiber, says Rindfleisch, "in the vast majority of cases these cells undergo a complementary infiltration with oil; they are converted into fat-cells." In caseous degeneration, on the contrary, it is not a conversion of the contents of the cell into oil, but a transformation in which fatty matter oocurs associated with various salts.

What is the cause of caseous degeneration? Why is it that in the one case a cell is transformed into a fat-cell—in the other into a caseous mass? A definite answer to this question is involved in the mystery of molecular action far beyond the eye of the microscope. The disease may be in the nucleus. For the present we can only in this matter do as the ancients did in so many—theorize; and a theory is not a bad thing if we do not forget that it is only a theory.

It seems to me that the cause of caseous degeneration has its origin in the scrofulus diathesis, or perhaps more properly, as the French sometimes term it, in lymphatisme. That caseous degeneration is always associated with inflammation of a chronic type is established. That scrofulous inflammations are almost invariably of a chronic kind is also decided. The association, then, of caseous degeneration as a result of the scrofulous diathesis is quite natural. What is the scrofulous or lymphatic diathesis? Says Billroth, this diathesis "exists chiefly in childhood. Persons with this diathesis, especially children, are greatly disposed to chronic inflammatory swellings of the lymphatic glands, even after inconsiderable irritations; to certain inflammations of the skin (eczema impetigo,) especially of the face and head; to catarrhal inflammations of the mucous membranes, especially of the conjunctiva, more rarely of the intestinal canal and respiratory organs; to chronic inflammations of the periosteum and of the synovial membrane of the joints. . . . The termination of the inflammation in caseous degeneration is not rare; it is particularly frequent in the lymphatic glands. Of course, it must have a very bad effect in the general nutrition where the mesenteric glands are degenerated in this way, and the chyle ducts thus mostly obstructed. Incurable atrophy of the entire body may thus be induced. The lymphatic diathesis is in most cases congenital, and is transmitted from generation to generation. But it may also be developed by improper modes of life. Among the most injurious causes are chief or exclusive diet of potatoes, flour, or sour bread, unhealthy, damp dwellings, lack of cleanliness, fresh air," etc.

From this picture, drawn by an accomplished hand, we perceive that in all morbific changes associated with the scrofulous diathesis, the lymphatic system and glands are especially involved. May it not then be that the entire series of results have for their primary origin an illy developed lymphatic system—a condition in which these vessels and glands are unusually delicate in structure as compared with other parts of the body, or as compared with others whose tissues are in every way normal. The analogies which may be drawn from other diseased or faulty conditions of the body certainly favors such a supposition. Cases of malformation or arrest of development of certain portions of the body are sufficiently frequent. Persons in whom the entire venous system is lacking in tone, who are disposed to varicose under slightly exciting causes, are met with; persons whose digestive system throughout its entire course is exceedingly irritable and easily deranged are frequently seen; and as to those who suffer from an exceedingly sensitive and easily deranged nervous organism, they need only be mentioned in this day.

Especially are we led to consider the plausibility of this view when we reflect on the fact that one of the special functions of the lymphatic glands and follicle is that of originating lymphatic corpuscles. If these glands and follicles are illy developed or perform their office in an imperfect manner, and, according to Billroth, are excessively irritable, how are we to expect healthy lymph, healthy blood, or healthy nutrition?

It is hardly necessary to refer to the fact that we have caseous degeneration arising in the course of other diseases—such as syphilis, glanders, typhoid fever, epithelial and fibroplastic tumors, etc. It does not follow that in these instances a conjoined lymphatism, hereditary or acquired, is not the predisposing cause of the caseous degeneration.

This view of the subject I know is not new, for Broussais long ago held that the local lesions of scrofula were due to irritation and inflammation of white tissues (tissues blancs) developed under the influence of lymphatic predisposition. It is not unusual, however, in medical science, that a proposition shall be posited and gain adherents in one generation, be rejected by the succeeding generation, and taken up and adopted by the third. We should not reject things only because they are old, or adopt them because they are new.

The close relation between caseous degeneration and lymphatism may further be inferred from the many experiments made in animals for the purpose of developing miliary tubercle by the inoculation of various substances. By these experiments it has been detirmined that certain animals are much more subject to caseous degeneration than others—especially is it so with the rabbit and guinea-pig. Now these animals seem to possess a more highly developed lymphatic system than others. Sections of the diaphragm of the rabbit are specially the beautiful network of lymphatic capillaries and serous canals, which are there so perfectly developed. Says Klein: "According to Ludwig and Schewigger-Seidel, we may distingush in the tendinous center of the diaphragm of the rabbit a system of wide lymphatics that lie perallel to one another, between the fibers of the radiating layer, and are lined by endothelium. . . . The plexus of the capillary lymphathics attains its greatest development at the posterior part of the centrum tendineum. In addition to these statements made by Ludwig and Schweigger-Seidel, it may be observed that in the rabbit, man, and cat, but especially in the guinea-pig, a rich system of serous canals and lymphatic lacunæ lined with endothelium can be demonstrated in the matrix itself, both on the abdominal and thoracic surface." While the excessive or peculiar development of the lymphatic system in certain animals, as thus indicated, does not prove a difference in texture, still it points in that direction; and when the field of comparative histology of the lymphatics shall be thoroughly explored, we will undoubtedly find that radical differences in quality do exist—differences which will account for the easy production of tubercle in one animal and its difficult production in another.

From this question, which is capable of indefinite expansion, I pass somewhat abruptly to the consideration of the relationship between caseous degeneration and tuberculosis.

Prof. Longworth, at the last meeting, gave a clear and graphic resume of the present state of knowledge on this subject, to which it would be impossible for me to add anything. He then showed that tuberculosis especially affect the lymphatics, but that as far as our knowledge goes there is nothing specific in tubercle—that not only will the insertion of tubercle beneath the epidermis of certain animals result in a general tuberculosis, which may be manifested in any part of the organism permeated by lymphatics, but that other substances of vegetable origin will produce the same result, provided always that at the site where the foreign substance is introduced, a primary focus of caseous degeneration occurs. We also learned that this result is obtained most frequently in certain animals, such as the rabbit and guinea-pig; whilst in others, such as the sheep, nothing of the kind follows either the introduction of tubercle, caseous matter, or foreign substances.

Now, the intimate relation which exists between caseous degeneration and lymphatism on the one hand, and on the other, between caseous degeneration and tuberculosis, leads me to believe that tuberculosis is nothing more than a peculiar manifestation of lymphatism or scrofulism. In other words, those persons or animals whose lymphatic vessels, glands, and follicles are of a peculiar delicate texture, be it hereditary or acquired, are just those in whom under favorable conditions and exciting causes tuberculosis will occur. In this I know I am asserting nothing new, for Lugol long ago maintained that tubercle is only a manifestation of scrofulous diathesis. And the distinguished Rokitansky expresses his opinion in these words: "For our own part we hold *tubercle* and *scrofula* to be *identical*—tuberculosis and scrofulosis to be one and the same disease.

A more recent writer, C. J. B. Williams, expresses himself in this way: "It is the scrofulous inflammation of lymphatic glands that makes them enlarge first, and then harden, and at length degenerate into caseous masses (instead of undergoing resolution or complete suppuration in healthy abscess,) which soften eventually and discharge from scrofulous sores. It is a similar type of inflammation which may develop miliary induration in the adenoid tissues of the lungs, tending in like manner to caseation, softening, and spreading, and to the formation of vomicæ."

The entire tendency of modern research, whether it be histological, pathologic, etiolcgic, or therapeutic, is to establish this fact.

Turn to the causes of lymphatism or scrofulism, and we find prominent among them a vagetable diet, damp dwellings, lack of fresh air, want of cleanliness, etc. Turn to tuberculosis, and we find the same predisposing causes enumerated. Look at the therapeutics of the disease, and we find cod-liver oil the remedy in either, par excellence.

It may be said, however, that the fact that a disease is disseminated by the lymphatics is no evidence that the cause of the disease is due to a morbid condition of those vessels. Truly, this is so. The lymphatics are absorbants, and thus syphilis enters and permeates every portion of the system; the lymphatic glands are enlarged and indurated—an evidence that the syphilitic virus is passing through them. Soon, however, this passes away, and these glands are to all appearances as before. We do not have a fire lighted up as in lymphatism or tuberculosis, which smolders and burns in the lymphatics themselves.

In conclusion, I may summarize by the following statements:

1. Caseous degeneration is a disease of cells in which for the protoplasm fat and salts are substituted.

2. Caseous degeneration differs entirely from fatty degeneration.

3. Caseous degeneration is an evidence of lymphatism or scrofulism in the individual or animal.

4. Tuberculosis is secondary to caseous degeneration.

5. Tuberculosis is only a manifestation of lymphatism or scrofulism.—*The Clinic.*

